# Improving Referral Adherence in a Developmental/Behavioral Clinic: A Quality Improvement Initiative

**DOI:** 10.1097/pq9.0000000000000853

**Published:** 2026-09-04

**Authors:** Sarah E. Rysdyk, Jamie L. Roberts

**Affiliations:** From the Division of Pediatric Psychology and Developmental Medicine, Department of Pediatrics, Medical College of Wisconsin, Wauwatosa, Wis.

## Abstract

**Introduction:**

Multiple studies have noted significant rates of emotional and developmental concerns in children, coupled with inconsistent rates of engagement with related services. Such inconsistency can lead to a range of negative outcomes. Our project aimed to increase the number of children who completed the intake process following referral to the Child Development Center from 57% to 80% by the end of the second quarter of 2022.

**Methods:**

The project team identified barriers to referral adherence and developed targeted interventions to address them. Plan-Do-Study-Act cycles included interventions such as moving the intake process to a phone call, direct outreach to families, enhancing education on Child Development Center services, and increasing communication with referring providers. We used control charts to assess the impact of interventions.

**Results:**

Completed intakes increased from 57% to 85% within 2 months and remained between 83% and 89% for the rest of the study. Referral completion rates for children younger than 5 rose from 45% to 75% or higher. The referral completion rate did not vary by ethnicity, race, or insurance type. Although increased referral completion rate led to increased wait times for appointments, 83% of patients who completed an initial intake followed through with an appointment at the Center.

**Conclusions:**

We used a quality improvement methodology to increase the percentage of patients who completed an initial intake following referral to the Center. The use of a phone intake option and consistent seeking of feedback and collaboration with all stakeholders involved in the project contributed to its success.

## INTRODUCTION

Access to healthcare for emotional and developmental concerns has been an issue that has garnered increased attention in the last few years, largely due to the growing demand for services. Nearly 20% of children and teens ages 3–17 years in the United States have a mental, emotional, developmental, or behavioral disorder, based on data from the 2022 National Healthcare Quality and Disparity Report.^1^ In the state of Wisconsin, where this project took place, data from the 2022–2023 National Survey of Children’s Health indicated that 27.4% of children, ages 3–17 years, reported having a mental, emotional, developmental, or behavioral problem.^2^ However, only 12.1% reported receiving treatment (in the form of medication) within the past 12 months. A recent study of children aged 2–5.5 years found that 23% met criteria for an emotional/behavioral disorder, and 22% qualified as having a developmental delay.^3^ Finally, a study, using data from the 2009–2010 National Survey of Children with Special Healthcare Needs, found 50% of children aged 0–4 years met criteria for a developmental delay, and 21.6% of these children had unmet therapy needs.^4^

These findings are consistent with past studies, which have found that a small percentage of referred youth receive a face-to-face visit with a mental health professional, with the lowest rates of referral completion ranging from 18% to 22%.^5,6^ A multitude of barriers to seeking help have been identified, some of which include concerns about financial obligations, inconvenience, confidentiality, stigma, and not knowing where to go.^7^ Research has also shown that, in pediatric settings, minority groups, families of lower socioeconomic status, patients with public health insurance, patients with longer wait times, and patients whose parents have a lower educational level or are younger are less likely to adhere to referrals.^8,9^ Conversely, families may be more likely to seek help with social encouragement, the availability of established relationships with professionals, and the identification of appropriate resources.^7^

The impact of not receiving treatment for emotional and developmental challenges can be significant for children and adolescents and their families. These challenges can include poor school performance and attendance, as well as declines in physical health, self-esteem, and social relationships.^10^ Increased family conflict and overuse of the healthcare system are also common consequences.^11,12^

### Study Aims

Consistent with the presented data, at the Child Development Center (CDC) of Children’s Wisconsin in 2021, 57% of referred patients completed the intake process after receiving a referral (the team considered a referral adhered to once the family completed a brief intake for the Center). Thus, this provider-led quality improvement (QI) project aimed to increase the rates of referral adherence. The specific aim was to improve referral adherence from 57% to at least 80% by the end of the second quarter of 2022. We selected this goal because it aligned with intake follow-through in other parts of the hospital’s mental and behavioral health system.

## METHODS

### Context

This QI initiative took place from January 2022 to February 2023 at the CDC of Children’s Wisconsin. The CDC is a multidisciplinary outpatient program that provides diagnostic evaluations and treatment for children aged 3 months to 18 years in a tertiary outpatient academic medical center. Although both diagnostic and treatment supports are provided at the Center, the focus of care and referrals coming into the Center is on diagnostics. Patients at the Center often display a complex presentation of neurodevelopmental concerns (attention deficit hyperactivity disorder, autism, intellectual disability, and significant learning disabilities) along with comorbid anxiety and mood struggles. Center providers consist of psychologists, speech and language pathologists, developmental pediatricians, and advanced practice nurse practitioners, who see patients from a wide referral region. The Center’s patient population consists of 60% privately insured children and 40% with Medicaid, with ages ranging from approximately 3 months to 18 years. In 2021, the Center served approximately 1621 children/families, of which the majority (67.5%) were White and 21.8% were Black/African American. In 2021, the Center received a total of 2,479 referrals. By 2022, our Center’s referrals had increased to 3,151, and in 2023, the Center received 3,730 referrals.

The hospital’s Mental and Behavioral Health (MBH) Access team was a critical piece of this QI initiative. The MBH Access team consists of individuals who speak with families interested in receiving services through Children’s Wisconsin to address their child’s emotional and/or developmental concerns. They engage with families via phone calls to gather background information on the child and information regarding their presenting concerns. This information is then used to support the appropriate triage of patients.

Due to the project’s aim to improve the quality of care for patients referred to the Center, the Children’s Wisconsin institutional review board approved the project as a QI project.

### Intervention

**Fig. 1.**
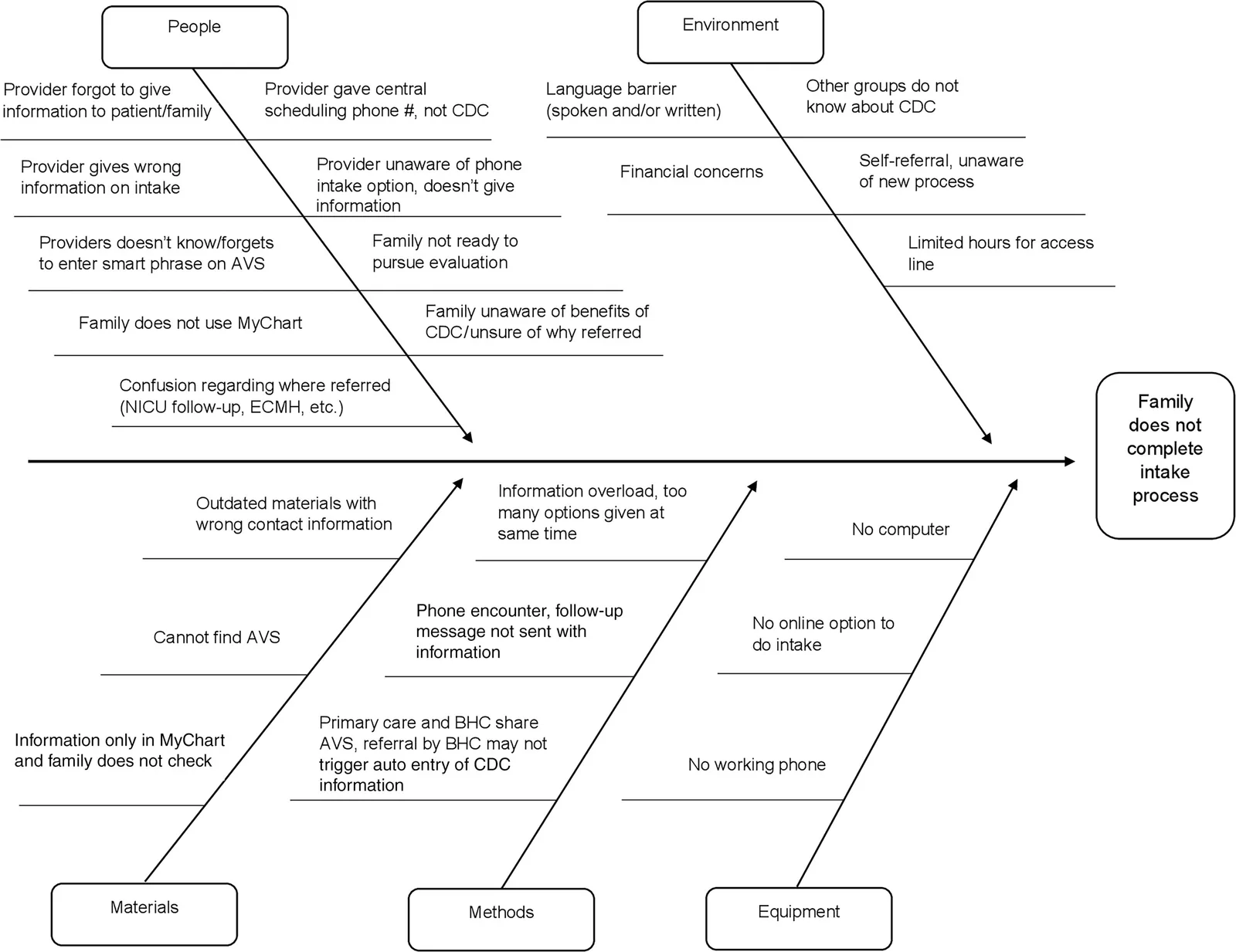
Fishbone diagram: barriers to patients adhering to referral to CDC. AVS, after-visit summaries; BHC, behavior health consultant; ECMH, early childhood mental health; NICU, neonatal intensive care unit.

**Fig. 2.**
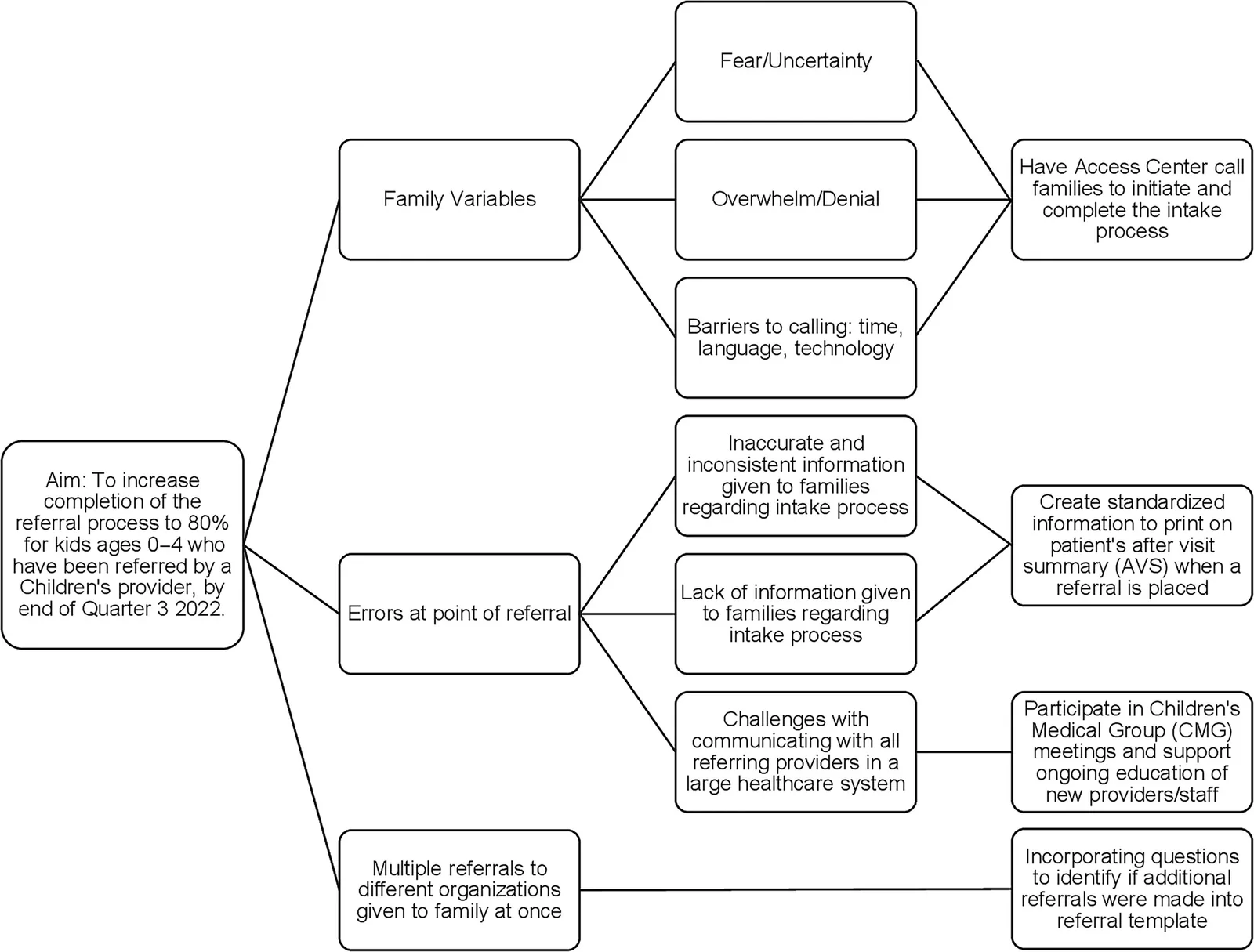
Driver diagram for intake adherence interventions.

An interprofessional team consisting of 2 psychologists (1 of whom served as the project lead), an advanced practice provider from the Center, the manager of the CDC, and a representative from the primary care group affiliated with the hospital began by developing a process map to outline the current referral completion process. Additionally, the team conducted chart reviews to gain a deeper understanding of what information families received about the Center and its intake process. The team also contacted patients who had undergone the process to learn about their experiences. These activities led the team to develop a fishbone diagram, outlining barriers to completing the intake process (Fig. 1). From there, they created a key driver diagram that outlined targeted interventions to make it easier for families to complete the intake process and improve the accuracy of information providers gave when referring families to the Center (Fig. 2).

The team conducted serial Plan-Do-Study-Act (PDSA) cycles to institute interventions and assess their impact (see Table 1). The first PDSA cycle’s intervention involved allowing families to complete the intake process via phone, by calling the MBH Access team (families were required to call the team, versus the team reaching out to them). In the past, the Center used paper forms, which were either mailed or emailed to families and then mailed or emailed back to the Center. (**See figure 1, Supplemental Digital Content 1** which describes referral process maps, https://links.lww.com/PQ9/A719.) The hospital’s MBH Access team increased personnel to support responding to calls from patient families referred to the CDC. During this phase, team leads held frequent meetings with the MBH Access leaders and team to ensure a smooth transition and address any questions that arose. The team also devoted significant effort to strengthening the connection between the CDC’s front desk staff and the MBH Access team by facilitating communication between the groups, ensuring the proper transfer of calls, fostering mutual understanding of roles, and building general trust.

**Table 1.** Project PDSA Cycles

PDSA Cycle	Cycle	Intervention Components
1	Intakes completed via phone	• MBH Access Center receives phone calls from referred patients and records information needed to complete their intake • Regular meetings are held with the Access team to answer questions, support their understanding of the CDC and its services, and check in to identify concerns • Supported communication between the CDC front desk staff and the Access Center to develop trust and a working relationship
2	Phone calls to patients younger than age 5 y	• MBH Access Center calls families of children younger than age 5 who were referred by Children’s Wisconsin primary care
3	Education of referring services and providers	• Conduct lunch and learn sessions with primary care and IBH providers to educate them on the CDC’s referral process • Develop a smart phrase, explaining the CDC’s referral process, for providers to use in communication with patients • Create educational opportunities for IBH providers to shadow at the CDC and learn about its services and referral process

Although the results from this initial PDSA cycle were extremely positive (data outlined in the Results section), the project team identified a select patient population, specifically children younger than 5 years referred by the Children’s Wisconsin primary care group, as continuing to exhibit lower-than-desired adherence to referrals. A second PDSA cycle ensued, involving members of the MBH Access team contacting the families of this select group of patients. Staff from the MBH Access team called families a total of 2 times during a period of 2–4 days. If they were unable to reach a family member, a voicemail was left requesting that they call back.

Given the high rates of miscommunication with patients at the time of their referral, a third and final PDSA cycle was conducted to improve communication between Children’s Wisconsin primary care and the CDC. This cycle ensured that accurate information was provided to families when they were referred to the Center. This was done in response to referring providers often providing inaccurate information on how to complete the Center’s intake process and what families should expect once a referral was placed with the CDC, as well as families being unsure of who they were referred to (see Fig. 1 for additional details). This cycle involved multiple educational interventions, including the implementation of a shadow experience during which integrated behavioral health (IBH) providers from the primary care setting visited the CDC to learn about services and establish open lines of communication between our groups. The Center also began conducting “Lunch and Learn” sessions with primary care and IBH providers to educate the group on MBH services at the hospital, with a focus on the CDC’s services. Finally, the team created smart phrases in the hospital’s electronic medical record, Epic (Epic Systems, Verona, WI), for providers to include in messages to families or after-visit summaries when referring a child to the Center. These phrases described the steps patients needed to take to complete their referral.

### Measures

The team used 2 primary measures: the rate of families who completed the intake process after being referred to the CDC, and the referral completion rates for children younger than 5 years of age referred by a Children’s Wisconsin primary care provider. They tracked patient information in a secure Excel database, which included variables such as the child’s age, ethnicity, form of insurance, and the time between referral and contact with the MBH Access team, among others. Weekly review of this database allowed the team to track the rate of referral completion.

Process measures included the number of families of children younger than 5 years of age that the MBH Access team reached and the number of times providers used the developed smart phrase when referring patients to the CDC. Aligned with our third PDSA cycle, we also tracked the number of views of our “Lunch and Learn” sessions and changes in the IBH providers’ understanding of the Center’s role in the mental and behavioral health system after having shadowed at the Center (based on pre- and postsurveys).

The team implemented balancing measures by tracking patient volumes, anticipating an increase in patients who completed the intake process and would subsequently require follow-up care at the CDC. The team also conducted monitoring of the rates at which those patients were scheduled and attended their appointments.

### Analysis

The team plotted the percentage of intakes completed over time on control charts. They applied standard probability-based rules to distinguish between common cause variance and special cause in both outcome and process measures. They also calculated summary statistics for patient variables, including age, ethnicity, and insurance status.

## RESULTS

The primary outcome, to increase overall referral completion rates to 80%, was met and exceeded, with completion rates consistently exceeding82%. Within 2 months of transitioning from a paper intake form to a phone intake process, we increased completion rates to 85%. We maintained them between 83% and 89% for the remainder of the data collection period (January 2022 to February 2023). During ongoing spot checks, we continue to maintain improved completion rates at or exceeding 82%. Overall, patient adherence to referral follow-through increased by 55% after transitioning from a paper to a phone intake process.

**Fig. 3.**
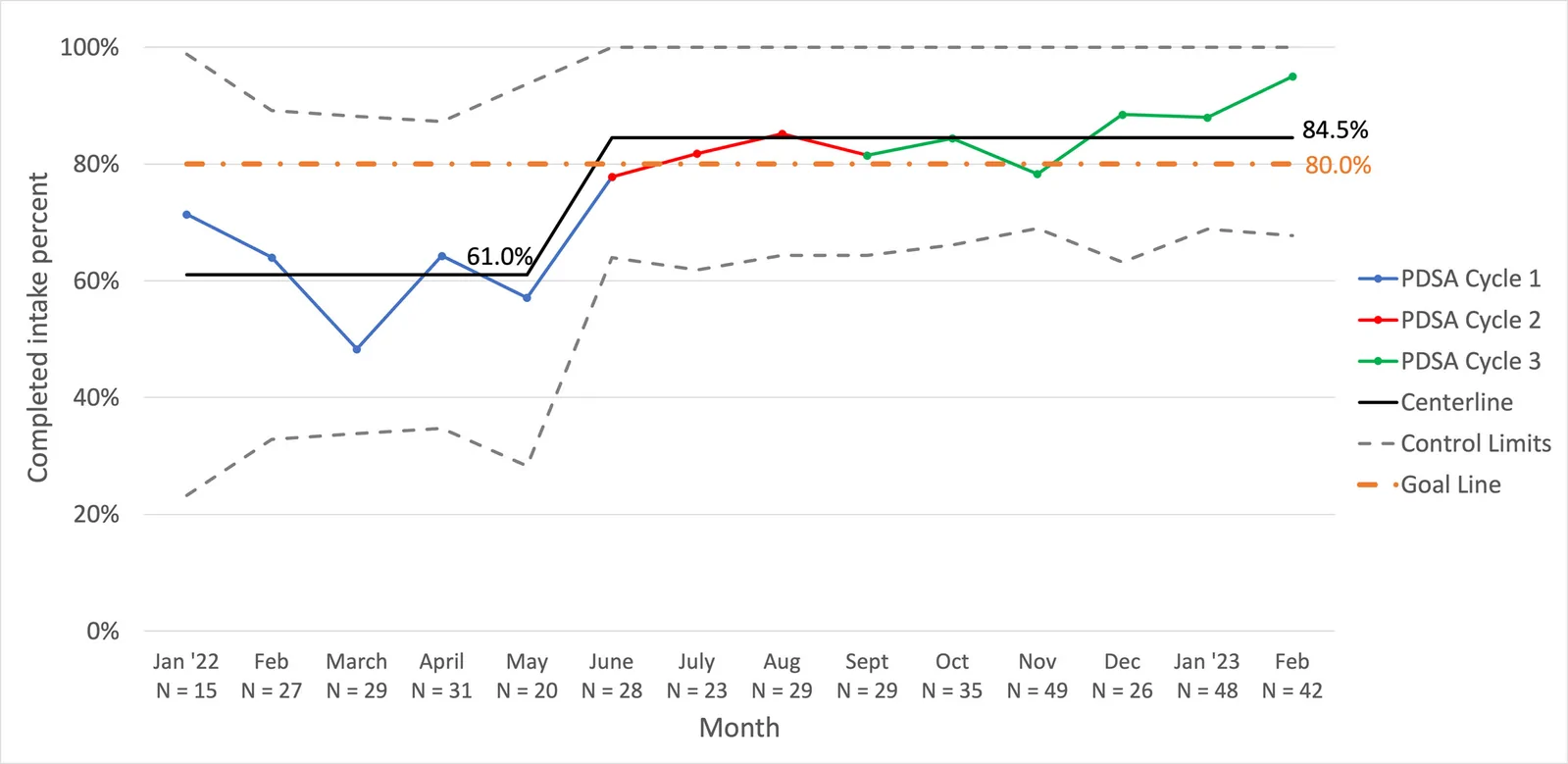
P-chart showing the percentage of intakes completed for patients ages 0–4 between January 2022 and February 2023.

Before the start of PDSA cycle 2, which included a call from the MBH Access team to the families of children younger than 5 years, the monthly referral completion rate for this age group was 45% (39 out of 86 referrals in January–April 2022). Completion rates for other age groups ranged from 100% (for patients 16 y and older) to 55% (for patients aged 5–6 y). Following the implementation of this intervention, the completion rate increased to approximately 75% or higher (Fig. 3). Notably, baseline data on referral completion rates, stratified by age, were not available before the commencement of this project.

Data from process measures, including the number of families our MBH Access team was able to reach when making phone calls to families of children younger than 5 years, showed high success in making direct contact. Specifically, the team made direct contact with 86% of the families called. The MBH Access team, which comprised 8 staff, hired 2 additional staff members to support this project and respond to the increase in phone calls associated with it. On average, a phone call with an MBH Access team member lasted between 15 and 20 minutes. Data analysis (see Table 2) revealed that the phone call referral completion option facilitated equitable access to care, as no significant differences were found in referral completion rates based on ethnicity, race, or insurance.

**Table 2.** Intake Completion Rates Based on Background Variables

		PDSA Cycle 1, January 2022 –May 2022, %	PDSA Cycle 2, June 2022–August 2022, %	PDSA Cycle 3, September 2022–February 2023, %	Overall Percentage
	N	Percentage of Completed Intakes			
Ethnicity					
Asian	121	85.4	94	94	92
African American	824	80.6	81.6	82	81
White	2,506	85.8	85.6	86.7	86
Native Hawaiian/Pacific Islander	6	100	N/A	75	83
American Indian/Alaskan Native	18	80	66.6	80	78
Blank	246	72.3	79.5	84	78
Insurance					
Commercial	1,735	87.5	86	87	87
Medicaid	1,921	80.8	84	84	83
Self-pay	4	100	100	100	100
Blank	51	70.6	71.4	68	67
Race					
Hispanic/Latino	272	55	56	72	64
Not Hispanic/Latino	1,063	67	65	75	71
Blank	49	68	57	65	65

Data related to improving communication between providers showed variable results. We conducted chart reviews to evaluate the use of a smart phrase that the team created to help providers share accurate information with families. Based on a review of 103 charts, 49% of referring providers provided information to families on the intake process, with only 28% of this information being accurate. Of the 28% who gave accurate information, only 34% used the created smart phrase. Tracking of providers’ access to “Lunch and Learn” recordings showed that 121 individuals and/or teams viewed the recordings for more than 4 months. Results related to IBH providers’ understanding of the CDC and the Center’s referral process showed improved knowledge with provider training opportunities (see Table 3).

**Table 3.** Pre- and Postsurvey Data From IBH Provider Training

Survey Area Assessed	Presurvey Mean	Postsurvey Mean
Understanding of how to make a referral to CDC	2.5	4.4
What to tell families when making a referral	2.1	4.4
Understanding of when to refer to the CDC	2.5	4.4
Comfort in determining a referral to CDC versus other similar clinics in the system	2.6	4.5

The 5-point Likert scale was used, with 2 response options depending on the question: option 1: 1 = terrible to 5 = excellent; option 2: 1 = extremely uncomfortable to 5 = extremely comfortable.

Data from balancing measures outlined an increase in the number of referrals received in our Center over time. This change constitutes a 50% increase in referrals over the course of 2 years. In response to this rise in referrals, as well as the increased adherence rates, our wait times for first appointments grew from 6–9 to 9–12 months. The rates of completed intake appointments for patients in 2022 yielded an approximate show rate of 83%.

## DISCUSSION

The growing demand for services to address emotional and developmental concerns in children is ongoing. Delayed access to services can lead to a host of negative outcomes, with pediatric settings making increased efforts to refer to specialized services. However, family, systemic, and procedural factors may negatively impact follow-through with referrals. Using QI methodology, the CDC improved our referral completion rate by incorporating a phone intake option. This transition was made even more successful by collaborating with support teams to initiate contact with families. This intervention supports other research that highlights the need for administrative procedures to improve systematic communication practices with patients, as well as the benefits of centralized intake practices for specialty referrals.^13,14^ Furthermore, this project demonstrates that there are tangible ways to improve follow-through with referrals, thereby increasing the chances of families accessing specialty care.

This project identified several interventions that supported our aim of increasing referral completion rates. Our data suggested that offering a phone intake option, rather than having families complete a paper intake form, led to a rapid and significant increase in our referral completion rates. However, this required advanced levels of collaboration with staff in the MBH Access Center, as well as a willingness to modify existing workflows. Shifting the established procedure of requiring families to call the MBH Access Center to have the team reach out directly to families led to further increases in referral follow-through. This shift supports research on increasing help-seeking behaviors, which outlines that having access to established and trusted professionals who can guide treatment options and next steps improves follow-through.^7^ By assigning a specific team member to reach out to families and offer an opportunity to disclose concerns, identify appropriate sources of help, and address related barriers, such as questions about cost, transportation, or confidentiality, we can improve follow-through with accessing offered services. In the current study, not only did we observe a significant increase in families completing the initial intake with our MBH Access Center, but these families also had a very high attendance rate for their initial appointment (83%), suggesting that positive engagement with our service staff may have facilitated the completion of appointments following referral.

Other interventions showed more varied levels of success. Process measures involved creating a smart phrase accessible to providers within the electronic medical record to encourage consistent information sharing with families about following up on specialty referrals, as well as providing access to a virtual and recorded discussion on practices for referring to the CDC. The team designed this intervention to help providers understand the specifics of the Center to which they are referring and support the dissemination of consistent information to families. However, our chart reviews suggested that educational opportunities with existing referring providers did not largely impact their referral practices. This finding is inconsistent with the literature, which suggests that primary care education programs that offer guidelines for standard referral processes improve the appropriateness of referrals.^14^ Although these supports may reinforce adaptive practices and the dissemination of information, inconsistent follow-through suggests that the continued use of phone intakes, particularly direct outreach to families, is more likely to impact referral completion. Educating new providers who interact in a supportive capacity with patients referred to the CDC at the point of hire may be more successful. This possibility is consistent with the literature, which suggests that allied health professionals offering direct referrals to specialists may streamline the referral process.^14^ Our efforts to provide information about our specialty clinic to IBH providers at the time of hire supported this possibility, as data showed that the providers’ level of understanding of what the Center offers and when (and how) to place a referral improved following our education with them.

There are limitations to our study that have had a direct impact on our Center. Notably, increasing referral completion rates has led to an even greater growth in our already expanding waitlists. As a result, families may be waiting longer for specialized services, which may impact long-term follow-through. This finding is critical, as we know faster access to intervention improves engagement in treatment and patient satisfaction.^13^ Additionally, the generalizability of the findings may be limited to systems that have the necessary staff to engage in established protocols, including making and receiving phone calls promptly, as well as a willingness to modify these protocols. Other hospital systems may lack personnel trained in behavioral health or the resources to establish and maintain a call center team to manage the influx of contacts with families. Finally, personnel and system-specific frameworks for disseminating knowledge may limit some of the collaboration and training efforts.

Although our aim was met and exceeded, our team is motivated to address the related impact of increased referrals and determine how this might affect patient follow-through with attending appointments, particularly given the increased waitlists. Future directions will involve examining the number of patients who completed the referral intake process, scheduled appointments, and showed up for their appointments once they came up for their turn on our waitlist. Exploring the previously mentioned data points will help validate whether a phone intake leads to higher rates of follow-through with specialty services. Research related to waitlist management and patient throughput suggests that standard practices for contacting patients to regularly review waitlists can help reduce wait times.^13^ Therefore, additional efforts will be made to evaluate our timeline and frequency of contact with patients on the waitlist. Given the variability of findings for best practices of educating referring providers, future interventions warrant additional data collection and evaluation to determine the most beneficial modes of collaboration and education. This intervention may include shifting the focus to training and supporting allied health professionals to ensure their understanding of appropriate and timely referrals, while exploring methods to increase their adherence to following through with recommended protocols.

## CONCLUSIONS

Our findings supported the use of a phone intake option to increase referral completion rates. To support the transition to this workflow and its ongoing operation, it was necessary to use and expand existing support within our system, namely the MBH Access team. Additionally, collaboration with referring providers and intake center staff was critical to the success of this project and continues to support the sustainment of this option. Providers can apply these processes to other specialty centers to improve follow-through with referrals.

## ACKNOWLEDGMENTS

The authors thank Jane McSweeney, APNP, Christopher Schwake, MD, and Lisa Meder, MBA, for their assistance with the study.

## Supplementary Material


