# Supplementary material for: Improving Referral Adherence in a Developmental/Behavioral Clinic: A Quality Improvement Initiative
Source: Pediatr Qual Saf. 2026 Sep 4;11(5):e853. doi: 10.1097/pq9.0000000000000853 (PMC13544776; doi:10.1097/pq9.0000000000000853)
Supplement: Supplementary file 1 [file pqs-11-e853-s001.pdf]

SDC, Figure 1
Referral Process Maps

Pre- PDSA Cycle 1 Process Map

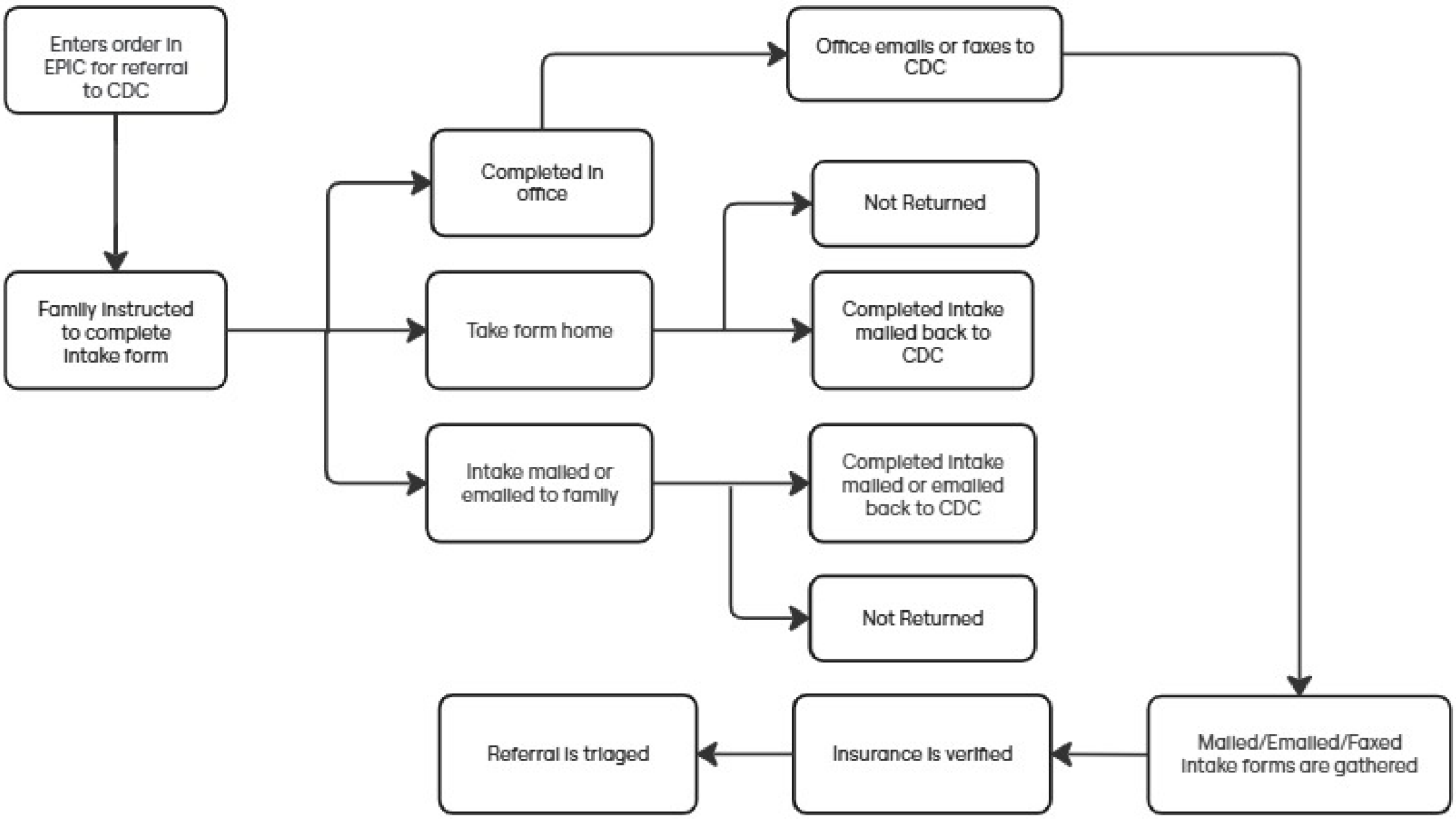

Final Process Map

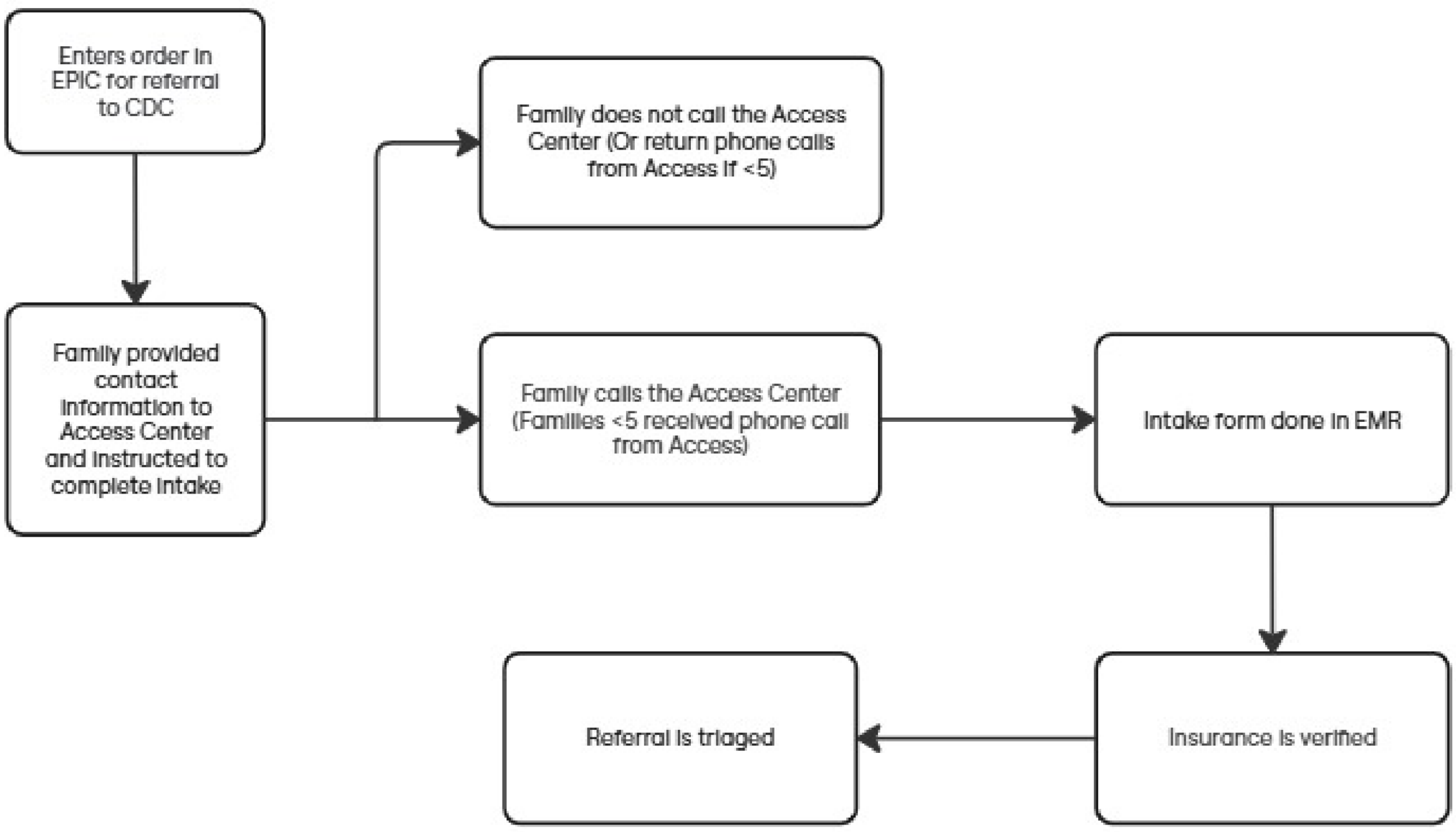

Referral Process Maps pre-PDSA Cycle 1 and final.

Improving Referral Adherence in a Developmental/Behavioral Clinic: A Quality Improvement Initiative. Rysdyk, S.
